# Localizing the position of the Segond fracture bed under CT measurements to determine the functional tibial insertion of an anterolateral ligament

**DOI:** 10.3389/fsurg.2023.1235750

**Published:** 2023-08-11

**Authors:** Ziteng Guo, Xuyang Wang, Guoshuai Liu, Yang Lu, Yuxi Bai, Jian Lv, Fei Liu

**Affiliations:** ^1^ Department of Orthopedics, The First Hospital of Qinhuangdao, Qinhuangdao, China; ^2^ School of Graduate, Hebei Medical University, Shijiazhuang, China

**Keywords:** anterolateral ligament, Segond fracture, rotatory instability, anatomy, computerized tomography

## Abstract

**Background:**

Many studies have confirmed the existence of ligament structures in the anterolateral region of the knee that maintain rotational stability of the knee joint, namely, the anterolateral ligament (ALL). Most scholars believe that knee joint reconstruction should be considered during revision surgery and a high level of pivot displacement test (stage 2 or 3). During ALL reconstruction, the choice of ligament reconstruction sites affects the success rate and prognosis of the operation. Therefore, the choice of ligament reconstruction sites is particularly important. There is little research on the lateral ALL tibia insertion point, and most clinicians use the midpoint Gerdy's tubercle and fibular head as insertion points. However, the reconstruction effect is not ideal.

**Objective:**

This study aims to measure the position of the Segond fracture bed on CT images to determine the ALL position of the tibia.

**Method:**

To determine the position of the Segond fracture bone bed, the CT AM Volume Share 2 system was used to manually measure the position of bone fragments in 23 Segond fracture patients. Using the highest point of Gerdy's tubercle in the CT axial slices and the outermost point of the fibular head in the CT axial slices as reference points, the direction and angle of the CT slices were adjusted to ensure that the highest point of the Gerdy tubercle, the outermost point of the fibular head, and the center of Segond fracture bed were in the same sagittal slice. A CT sagittal slice measures the vertical distance from the center of the Segond fracture bed to the Gerdy-fibular line segment (G-F line segment), which is the line connecting the highest point of the segment to the outermost point of the fibula. The distance from the vertical point at the center of the Segond fracture bed of the G-F line to the highest point of the Gerdy tubercle was measured. All measurements were performed using the same measurement standard and were expressed as a percentage of the length of the G-F line. The measured results were statistically analyzed using SPSS 25.0 descriptive statistical research methods.

**Results:**

The average length of the G-F segment measured on CT images was 39.6 ± 2.0 mm, and the average vertical length from the center of the Segond fracture bed to the G-F segment was 13.1 ± 1.1 mm, accounting for 33.2% ± 2.1% of the length of the G-F segment. The length from the vertical point of the fracture bed on the G-F line segment to the highest point of the Gerdy tubercle was 14.7 ± 1.3 mm, accounting for 37.1% ± 2.9% of the length of the G-F segment.

**Conclusion:**

Through the study of the CT measurement of the Segond fracture location, we obtained the location of the functional tibial insertion of ALL, which is different from the anatomical insertion of ALL and is more inclined to the Gerdy tubercle and above, which has reference value for the treatment of recovering the function of anterolateral ligament after reconstruction.

## 1. Introduction

The anterolateral ligament (ALL) was first described by Paul Segond, who reported a “pearlescent, highly resistant fibrous band” exhibiting extreme tension under excessive internal rotation (1). Since then, the anatomy and function of the anterior lateral ligament have been the focus of scholars' research. In 2007, Vieira et al. officially named it the anterior lateral ligament (2). Subsequently, many scholars have confirmed that the ALL can maintain rotation and anteroposterior stability of the knee joint and have proposed that most anterior cruciate ligament injuries are accompanied by anterior lateral ligament injuries (3–6). Therefore, scholars have proposed that surgical repair should be performed on ALL while reconstructing the anterior cruciate ligament (ACL) to reduce the pivot displacement of the knee joint in patients. Compared to the selection of the femoral insertion of the ALL, there is less controversy for the tibial insertion, which is in the middle of Gerdy's tubercle and fibular head (7). However, some ALL-class isometric studies have found that even the less controversial tibial insertion is not a perfectly isometric point (8–10) when paired with various types of femoral attachment points for reconstruction. Claes et al. recently used statistical analysis to prove that Segond fractures are bone avulsions caused by forces acting on the tibial insertion point (11). Therefore, the functional tibial insertion of ALL can be determined by measuring the position of the Segond fracture bed.

## 2. Materials and methods

### 2.1. Selection criteria of patients

Between March 1, 2012 and March 1, 2022, 2,000 patients diagnosed with ACL injury in the Radiology Department of the First Hospital of Qinhuangdao were examined with CT and CT 3D imaging. All corresponding CT and CT three-dimensional imaging data were manually examined to determine the presence of the Segond fracture (avulsion fracture of the proximal lateral tibia), and the medical records were reviewed to determine the time between the injury and radiological diagnosis of the Segond fracture. We studied the hospital medical records and medical imaging scans of these patients and then analyzed the CT images of these knees to evaluate the size, shape, direction, and degree of displacement of Segond fracture fragments, as well as radiological evidence of related bone and soft tissue injuries. Using Volume Viewer in the AM Volume Share 2 system, we measured the width of the torn bone mass and screened images of Segond fracture fragments with a bone block width of less than 11.3 mm, excluding small or unclear bone fragments. Finally, 23 Segond fracture images that met the above conditions were selected. Table 1 summarizes the information data for each patient.

**Table 1:** Anthropometric data for the patients.

Knee	Sex	Age, years	Side	Weight, kg	Height, m
1	W	49	L	52	1.63
2	W	34	R	62	1.65
3	M	36	L	82	1.75
4	M	52	R	78	1.72
5	M	54	R	75	1.70
6	W	56	L	68	1.65
7	M	38	L	85	1.78
8	M	18	L	72	1.76
9	M	13	L	60	1.67
10	M	20	L	83	1.82
11	W	75	R	54	1.62
12	W	68	L	59	1.66
13	W	22	L	68	1.67
14	M	31	R	87	1.85
15	W	31	R	66	1.65
16	W	53	R	55	1.60
17	W	56	L	59	1.62
18	W	56	L	64	1.65
19	W	61	R	51	1.62
20	W	64	R	63	1.67
21	M	13	L	40	1.58
23	W	28	R	87	1.80
Mean ± SD		42.2 ± 18.2		66.8 ± 12.7	1.69 ± 0.1

### 2.2. Selection of reference points

**Figure 1:**
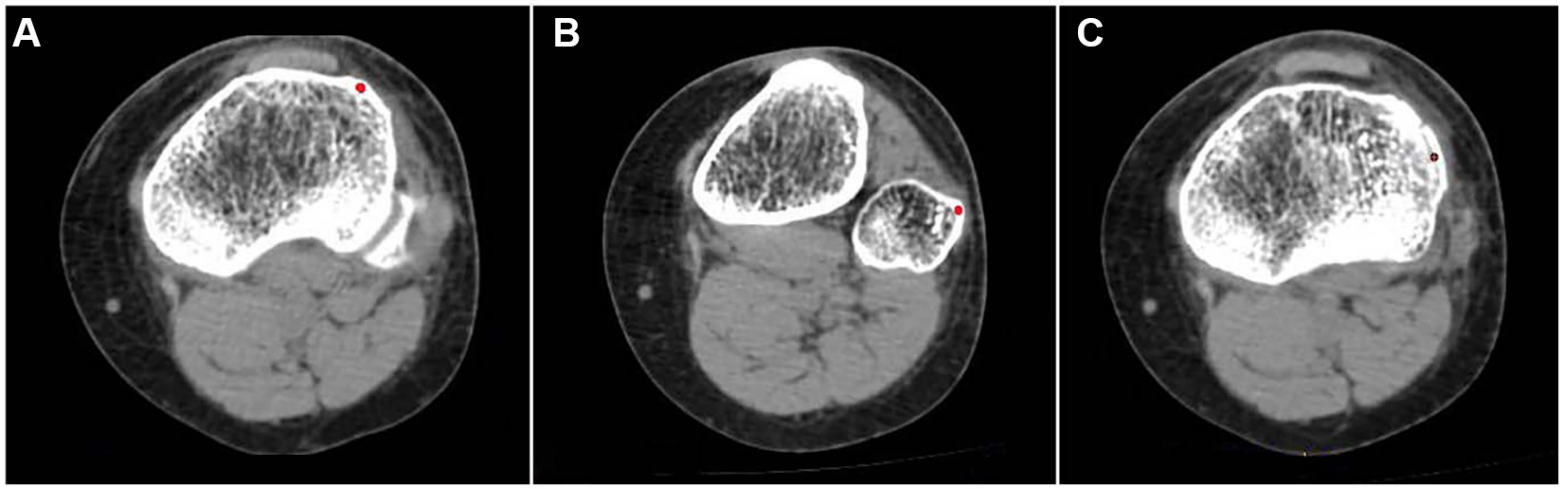
(**A**) Highest point of the Gerdy tubercle in the CT axial slices. (**B**) Outermost point of the fibular head in the CT axial slices. (**C**) Center of the Segond fracture bed in the CT axial slices. Red dots indicate reference points.

To facilitate palpation and identification by clinicians, we selected the highest point of the Gerdy tubercle in the CT axle section and the outermost point of the fibular head in the CT axle section as reference points for localization. In locating the fracture fragment, the midpoint of the Segond fracture fragment in the axle position was selected. The position of the red dot is shown in Figure 1.

### 2.3. Measurement methods

**Figure 2:**
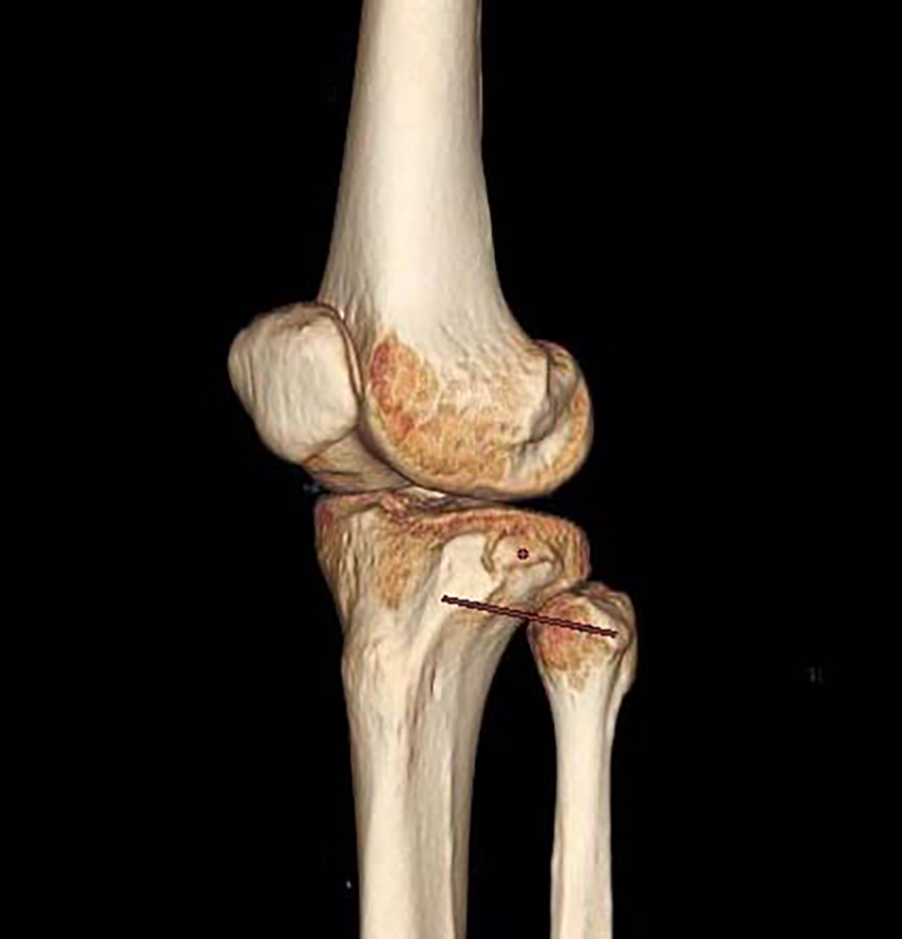
3D reconstruction image of the knee in lateral view.

**Figure 3:**
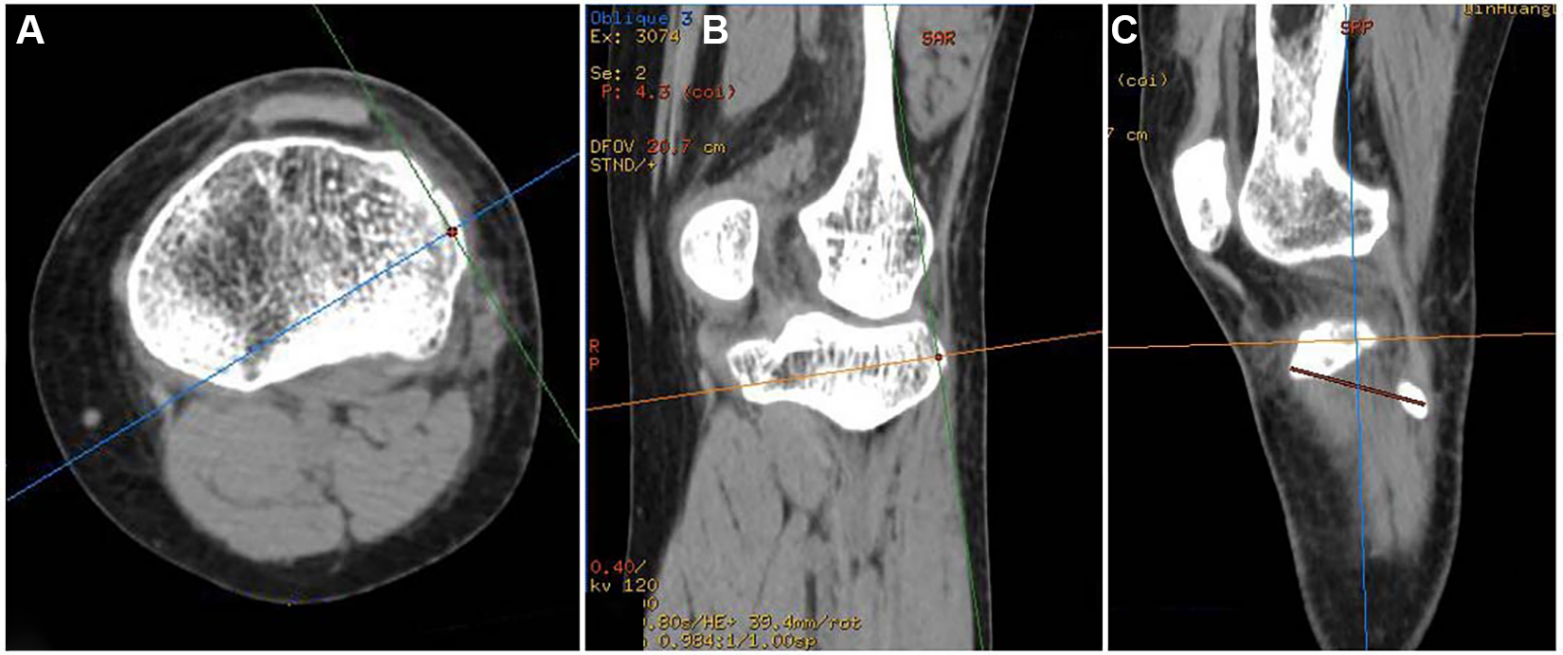
(**A**) Cross line cutting direction at the center of the bone bed in the axial slices image. (**B**) Cross line cutting direction at the center of the bone bed in a coronal image. (**C**) By adjusting the direction of cross line cutting at axial and coronal positions, the center of the bone bed appears on the same sagittal slices as the G-F line.

**Figure 4:**
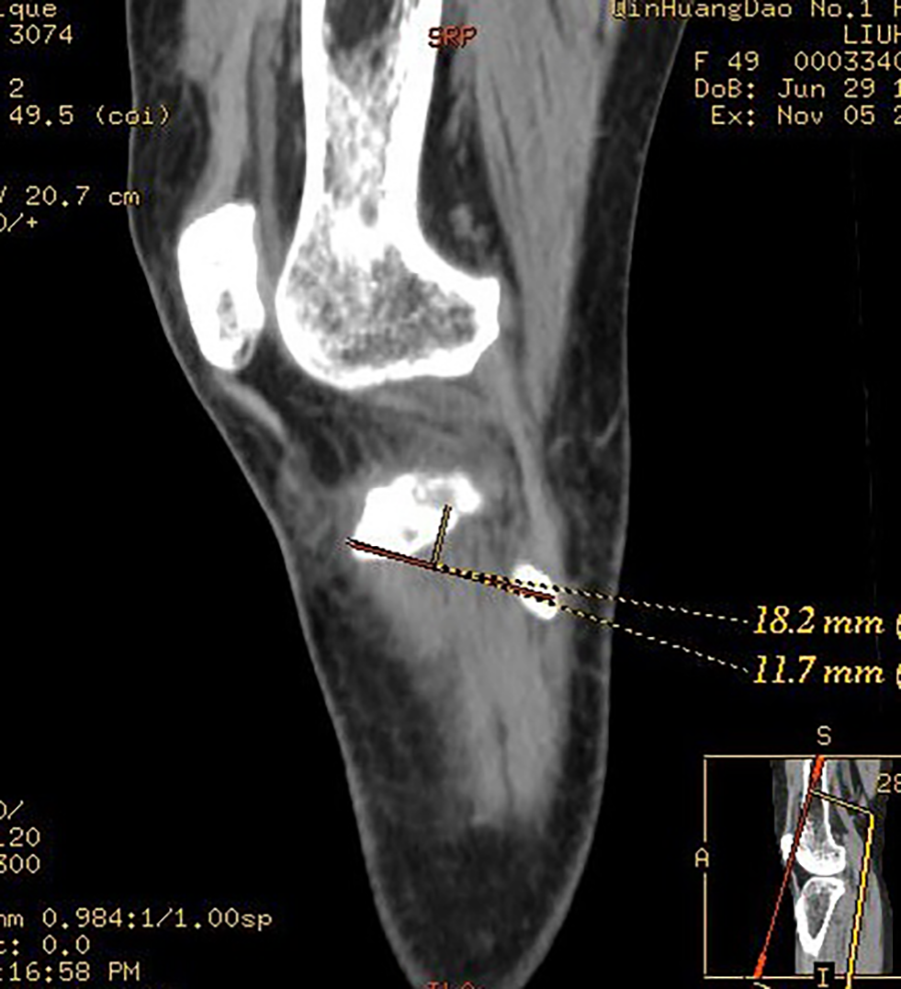
Measurement of the length from the Gerdy tubercle to the vertical point of the Segond fracture bed at the G-F line segment and the length from the vertical point to the midpoint of the Segond fracture bed.

To describe the position of the bone bed in Segond fractures, we used the Volume Viewer “Position Cursor” tool of the AM Volumetric Shared 2 system. We created a positioning point at the highest point of the Gerdy tubercle on the axial slices, which synchronizes with the 3D reconstruction image. We used the same method to create a second anchor point at the outermost point of the fibular head. The two points were displayed synchronously in a 3D image reconstruction and connected to a line segment, which we call the Gerdy-fibular line segment (G-F line segment) (Figure 2). In normal CT sagittal images, the highest point of the Segond fracture fragment, the highest point of the Gerdy tubercle, and the outermost point of the fibula were not on the same plane, making two-dimensional measurement impossible. We needed to manually adjust the slice angle of the sagittal image so that these three points were on the same plane of the image. The specific method is as follows: rolling the axial slices determines the central point of the Segond fracture bed and establishes a cross line (two cutting lines perpendicular to each other) based on the central point of the Segond fracture bed. At this time, a cross line appeared simultaneously at the center of the sagittal and coronal bone beds. We adjusted the direction of the cut line 360in the axial and coronal images, cut at different angles, and observed the sagittal image until the G-F line appeared completely in the sagittal image (Figure 3). To ensure the complete appearance of the G-F line segment in the sagittal position of the image, we needed to verify this. We created a new positioning point, moved the new positioning point in the sagittal image so that it overlaps with the two endpoints of the line segment, and observed the 3D reconstruction. If this point overlaps with the highest point of the Gerdy tubercle and the outermost point of the fibular head in the three-dimensional reconstruction, we can determine that this line segment is the G-F line we are looking for. We measured the length of the G-F line using the 2D measurement tool in the system and made a perpendicular line from the midpoint of the Segond fracture bed to the G-F line segment. We measured the length from the Gerdy tubercle to the vertical point of the Segond fracture bed at the G-F line segment and the length from the vertical point to the midpoint of the Segond fracture bed (Figure 4). All parameters were measured as a percentage of the length of the reference G-F segment. This represents the position of the functional insertion on the tibial side of ALL. The measurements were taken by two authors at least 30 days apart. The final reported value is the average measured by two observers. The measurements were analyzed using descriptive statistics, and the mean, median, minimum, maximum, and standard deviation were analyzed using SPSS 25.0 software.

## 3. Results

**Figure 5:**
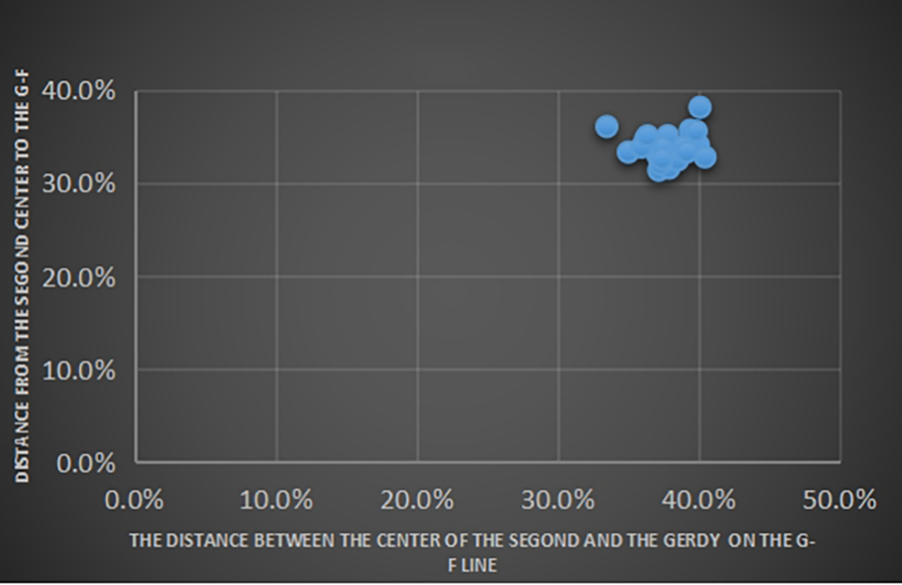
Measurement of the *x*-axis and *y*-axis as a percentage and given in G-F dimensions.

The average length of the G-F segment measured on CT images was 39.6 ± 2.0 mm, and the average vertical length from the center of the Segond fracture bed to the G-F segment was 13.1 ± 1.1 mm, accounting for 33.2% ± 2.1% of the length of G-F segment. The length from the fracture bed vertical point on the G-F line segment to the highest point of the Gerdy tubercle was 14.7 ± 1.3 mm, accounting for 37.1% ± 2.9% of the length of the G-F segment (Table 2). We took the highest point of the Gerdy tubercle as the origin and the direction of the G-F line segment as the horizontal axis. We took the line passing through the Gerdy tubercle in the vertical direction of the G-F line segment as the longitudinal axis. We established a scatter plot to represent the position of the center point of the Segond fracture bed (Figure 5).

**Table 2:** CT measurements of the Segond fracture.

	Mean	Median	Minimum	Maximum	SD
G-F line segment, mm	39.6	39.0	36.2	43.7	2.0
Gerdy tubercle to vertical distance, mm	14.7	14.7	11.7	17.1	1.3
Segond fracture central point to the G-F line segment, mm	13.1	13.2	10.9	15.1	1.1

## 4. Conclusion

Through the study of the CT measurement of the Segond fracture location, we obtained the location of the functional tibial insertion of ALL, which is different from the anatomical insertion of ALL and is more inclined to Gerdy tubercle and above, which has reference value for the treatment of recovering the function of anterolateral ligament after reconstruction.

## 5. Discussion

ACL injury is the most common sports injury of the knee joint. Through the surgical reconstruction of the anterior cruciate ligament, the postoperative motor function of patients was restored to a great extent. However, the progress of some cases is still not satisfactory (12, 13). The cause of ACL rupture is usually compound violence, often combined with damage from other knee stabilization devices. Although our knowledge and surgical ability to restore ACL anatomy and function have improved, approximately 1.7%–7.7% of patients still experience unstable ACL rotation and failure (7). Claes et al. confirmed that 78.8% of patients with ACL rupture had anterior lateral ligament injury (1). This has led the orthopedic community to reconsider ALL for restoring knee joint stability, and new technologies have emerged for ALLR (Anterolateral ligament reconstruction) (12). The combination of ACL reconstruction and ALL reconstruction during ACL reconstruction can preserve rotation and anteroposterior stability of the knee joint of patients. However, similar to ACL reconstruction, determining the most suitable reconstruction site is crucial for ALL reconstruction surgery, and the selection of sites often determines the success rate of the surgery and the patient's prognosis (14). At present, in surgical ALL reconstruction, most physicians choose the anatomical insertion point of the ALL tibia, which is the middle of the Gerdy tubercle and fibular head, as the tibial side for ALL reconstruction. However, this site is not the perfect tibial insertion we want. The result may be caused by two reasons. On the one hand, the study found that ALL is a non-isometric ligament, and the variation of the attachment morphology of this ligament is high, so simply fixing the ligament on the anatomical insertion could not achieve the isometric effect of the reconstructed ligament after surgery, resulting in the change of ligament tension during the extension and flexion of the knee joint (15–19). On the other hand, ALL is a ligament structure that starts from the lateral femoral condyle, runs forward and down between the lateral collateral ligament and the popliteal tendon, bifurcates in the middle, and ends at the lateral meniscus and the lateral tibial condyle (20). The ALL anatomic insertion of the meniscus is often ignored during reconstruction. At the same time, because of the anatomical characteristics of the meniscus and when the range of motion of the knee is different, the meniscocapsular complex will be subjected to varied tension and pressure condictions (21, 22); it is difficult to fix the ligament on the meniscus. Therefore, we believe that there may be a functional insertion on the tibial side with more concentrated compound stress, which is different from the anatomical insertion.

With the recognition of the existence of ALL, many scholars began to explore the stress of ALL on the tibial side. Claes et al. proved by statistical methods that the position of the Segond fracture fragment is the same as that of bone avulsion in ALL (11). Subsequently, Porrino et al., through the identification of ALL on MRI, observed that the tibial insertion of ALL is attached to the Segond fracture bone fragment (23). Later, a more detailed analysis showed that the Segond fracture fragment is the avulsion of the trabecula of the lateral tibial plateau caused by the traction force during ALL injuries (24). When ALL is torn, the position of the Segond fracture fragment is more likely to reflect the comprehensive stress on the tibial side. Therefore, we believe that the position of the Segond fracture avulsion can be used as the force-bearing point of ALL on the tibial side. This force-bearing point is different from the anatomical insertion. We believe that the functional insertion is a simulated insertion on the tibial side, which integrates the joint stress of the anterolateral ligament on the tibial side and reconstructs the ligament at this insertion, which may better restore the function of ALL on the rotational stability of the knee joint.

We measured the position of the Segond fracture bed to determine the position of the tibial functional insertion of ALL. We used the Volume Viewer in the AM Volume Share 2 system to measure the position of the bone bed. Based on the tibial attachment area of ALL of 11.3 ± 2.8 mm (1), we screened patients with Segond fractures with bone fragments smaller than or equal to 11.3 mm to avoid inaccurate force points caused by oversized bone fragments. When selecting the position reference point, we consider the convenience and accuracy of the surgeon's palpation during the surgery. We choose the highest point of the Gerdy tubercle in the cross section and the outermost point of the fibular head as the reference points. To achieve the accuracy of two-dimensional measurements, we determined the highest point of the Gerdy tubercle and the outermost point of the fibular head on the CT axial slices and connected these two points into a line segment called the G-F line segment. If the midpoint of Segond fracture bone fragments does not pass through this line segment, then the midpoint of Segond fracture bone fragments and the G-F segment lines can determine a plane. We use the midpoint of the Segond fracture bed as the reference point to cut along two perpendicular lines in each direction of the coronal and sagittal images. When the midpoint of Segond fracture bone fragments and the G-F segment line appear simultaneously in the sagittal image, two-dimensional measurements can be performed on that plane.

The measurement result of this experiment is that the tibial functional site of ALL is 37.1% ± 2.9% from the highest point of Gerdy's nodule in the direction of G-F segment and moves up 33.2% ± 2.1% in the direction of the vertical G-F segment. It can be seen that the results of this study are significantly different from the position of the traditional anatomical insertion (the midpoint of the line between the Gerdy tubercle and fibular capitulum). This result confirms that the point of force on the tibial side of ALL is the functional insertion for the joint action of structures outside the joint capsule. Thus, the position of the insertion point is not the commonly used anatomical point before. The reason may be that when the anterolateral structure of the knee joint is under stress, it is not the result of a structure of the anterolateral ligament, and the joint force of the surrounding joint capsule and meniscus plays a role in the anterolateral structure, so the point of force concentration is not at the anatomical point. This point is more inclined to the Gerdy nodule and is upper than the anatomic insertion.

Long before ALL was recognized by most people, scholars put forward the concept of the anterolateral structure, which is a compound anatomical structure of the anterolateral knee joint that mainly controls the rotation and stability function of the knee joint (25). Later, LET (lateral extra-artificial tenosis) was proposed. The principle of LET controlling the rotational relaxation of the knee joint is not to reconstruct a well-defined ligament but to limit the excessive internal rotation of the tibia (26). LET operation includes Lemaire operation, modified Lemaire operation, and Macintosh operation. Both are reconstruction techniques that use the iliotibial tract as a graft and retain its distal attachment point in the Gerdy tubercle. This method is also called functional reconstruction, and its reconstruction scope includes all anterolateral structures that affect knee rotation, the superficial layer of the iliotibial tract and iliopatellar tract, the deep layer of the iliotibial tract, the anterolateral ligament, and the anterolateral joint capsule (27, 28). We support the idea of integrating a force point and reconstructing the ligament in all lateral structures of the knee joint. However, we prefer to use ALL as the main body in the lateral structure of the knee joint to find the functional force point of the tibial side of ALL. Because many scholars have confirmed that ALL plays a major role in the rotational stability of the knee joint, the study found that when the knee flexion angle is greater than 35°, the internal rotation limit mainly depends on the anterolateral ligament of the knee (29). However, because ALL is relatively thin and the load tension of the ligament is small through anatomical study, the stress of ALL is easily affected by the surrounding structure when the knee joint rotates (30), resulting in the difference between the functional insertion of ALL on the tibial side and the anatomical insertion of ALL. We believe that the knee injury is a compound injury of the joint capsule and ligament. It may be inappropriate to simulate the reconstruction of the anatomical structure of ALL simply. It is necessary to consider the factors of compound stress. The proper anterior and upward movement of the tibial side of ALL may play a better role in the reconstruction of ALL.

The innovation of this study is to quantify the tibial functional sites of ALL by CT measurements, which confirms that the functional sites of ALL are different from the anatomical sites and provides a new choice for the tibial construction sites of ALL during operation. When measuring the midpoint position of the Segond fracture bed, we choose the length of the G-F segment as the measurement unit and mark the position as a percentage of the length of the G-F segment. This can reduce the error in patient height and weight while also facilitating intraoperative measurement by the surgeon. This article proposed the concept of functional insertion and successfully confirmed that its location is different from the anatomical point, suggesting that the ligament structure of the knee joint and even other joints, especially the structure outside the joint capsule, is not a simple connection of two points and one line but should be a functional complex, which has implications for scholars to further study the structure outside the joint capsule. In this experiment, by analyzing the results of ligament damage, we can reverse understand the compound effect of ligament structure and measure its position, which can be an innovative method for studying knee joint ligaments and has certain significance.

However, this experiment also has some limitations: first, the sample of three-dimensional CT reconstruction of Segond fractures is small (*n* = 23), which may lead to inaccurate measurement results due to the small number of measurement samples. Second, this study only proposed the theory of the tibial functional insertion point of ALL, which provided a new choice for clinical reconstruction of the tibial side of ALL but did not verify the clinical effect of ligament reconstruction at this site. In clinical surgery, this site is closer to the articular surface than the traditional site. When fixing the ligament, a minor injury occurring in articular cartilage may lead to progressive injury and degeneration (31). The fixation method and the size of the fixation during reconstruction need to be further studied.

## Data Availability

The original contributions presented in the study are included in the article/supplementary material, further inquiries can be directed to the corresponding author.
